# Field application of nanoliposomes delivered quercetin by inhibiting specific hsp70 gene expression against plant virus disease

**DOI:** 10.1186/s12951-021-01223-6

**Published:** 2022-01-04

**Authors:** Jie Wang, Kaiqiang Hao, Fangfei Yu, Lili Shen, Fenglong Wang, Jinguang Yang, Chenyu Su

**Affiliations:** 1 Key Laboratory of Tobacco Pest Monitoring Controlling & Integrated Management, Tobacco Research Institute of Chinese Academy of Agricultural Sciences, Qingdao, 266101 China; 2 College of Plant Protection, Shenyang Agricultural University, Shenyang, 110866 China

**Keywords:** Plant virus disease, Quercetin, Nanoliposomes, Field application

## Abstract

**Background:**

The annual economic loss caused by plant viruses exceeds 10 billion dollars due to the lack of ideal control measures. Quercetin is a flavonol compound that exerts a control effect on plant virus diseases, but its poor solubility and stability limit the control efficiency. Fortunately, the development of nanopesticides has led to new ideas.

**Results:**

In this study, 117 nm quercetin nanoliposomes with excellent stability were prepared from biomaterials, and few surfactants and stabilizers were added to optimize the formula. *Nbhsp70er-1* and *Nbhsp70c-A* were found to be the target genes of quercetin, through abiotic and biotic stress, and the nanoliposomes improved the inhibitory effect at the gene and protein levels by 33.6 and 42%, respectively. Finally, the results of field experiment showed that the control efficiency was 38% higher than that of the conventional quercetin formulation and higher than those of other antiviral agents.

**Conclusion:**

This research innovatively reports the combination of biological antiviral agents and nanotechnology to control plant virus diseases, and it significantly improved the control efficiency and reduced the use of traditional chemical pesticides.

**Graphical Abstract:**

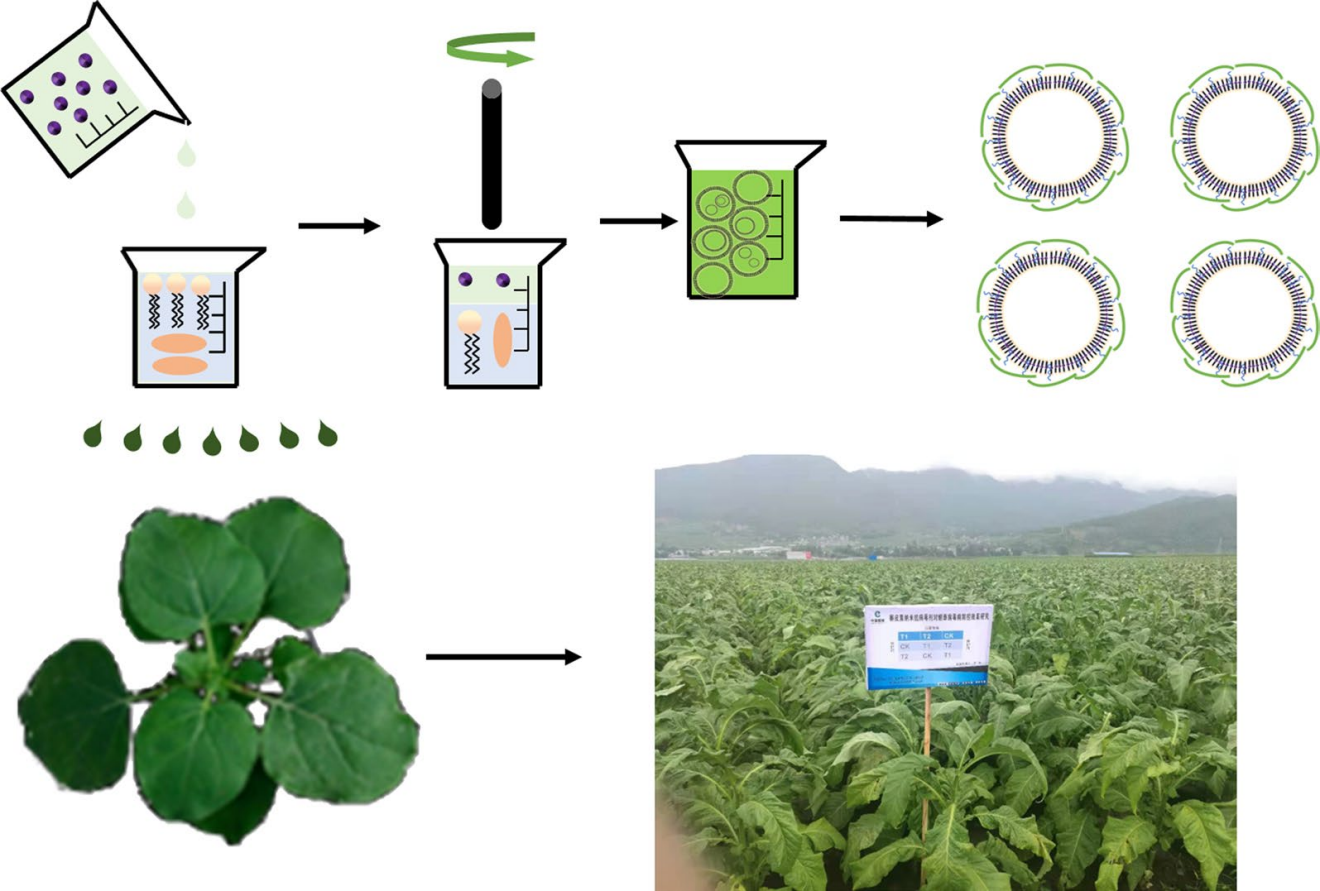

**Supplementary Information:**

The online version contains supplementary material available at 10.1186/s12951-021-01223-6.

## Introduction

Plant virus diseases known as plant cancers, accounting for nearly half of all plant diseases [1–3]. Plant viruses affect 118 genera and 1516 species in 26 families [4]. Plant viruses cannot infect humans and other higher animals, however, once the main crops are infected by plant viruses, the yield and quality will be seriously affected [5, 6]. According to statistics, economic losses caused by tomato spotted wilt virus alone exceed $1 billion per year [7], while another two single-stranded DNA viruses, tomato yellow leaf curl virus and African cassava mosaic virus, cause $1.9–2.7 billion losses [8]. The most important plant virus worldwide, tobacco mosaic virus (TMV), cause plants to fail to grow normally because of leaf tissue necrosis [9, 10]. Research on TMV dates back to 1901, and in 1946, Stanley won the Nobel Prize in Chemistry for the elucidation of the molecular structure of TMV. Billions of dollars are spent each year to prevent and control plant virus diseases [11]. In crop production, the major precautions to prevent plant virus diseases are as follows: growth of a virus-free propagule [12], induction of cross protection [13] and development of virus-resistant plants by editing genes and attenuated vaccines [14, 15]. However, once a plant is infected with viruses, antiviral agents, which can effectively reduce the impact of infection on the growth of the host plants, become the most important protection measure [16]. In recent years, due to their environmentally friendly natural, natural antiviral agents have been widely used in the control of plant virus diseases.

Quercetin (3,5,7-trihydroxy-2(3,4-dihydroxyphenyl)-4H-benzopyran-4-ktone) is a flavonol found widely in plant [17, 18]. It is widely used in the fields of food and medical treatment because of its antioxidant function [19, 20] and antitumor activity and its capacity to inhibit a variety of liver and influenza viruses [21]. Similarly, many studies have reported its inhibitory effect on various plant viruses [22], hence, based on its low cost, quercetin has considerable potential for control of plant virus diseases. For example, Wang found that 1 mol/L quercetin could effectively inhibit the proliferation of cucumber necrosis virus (CNV), turnip crinkle virus (TCV) and TMV in leaf cells of the model plant *nicotiana benthamiana* (Nb), and the inhibition rate to viral RNA was up to 90% [23]. As a planar molecule with compact stacking structure and strong intermolecular attraction, quercetin would not be dissolved by solvents [24]. Moreover, quercetin and its derivatives are prone to degrade under natural conditions [25], although quick degradation of active ingredients means an advantage for the environment and nontarget organisms, it reduces the control efficiency for plant virus [26]. Therefore, there is an urgent need to develop a carrier with excellent solubility and stability to deliver quercetin.

In recent years, nanotechnology has been actively utilized in various fields, especially in nanomedicine [27, 28], nanobiology [29, 30], etc. In particular, the active ingredients of hydrophobic drugs for the treatment of cancer have been encapsulated via nanotechnology: the biocompatibility of nanoparticle promoted the transport of foreign materials through the cell membrane, and by modifying their surface, the targeting ability of the delivery system will be enhanced [31]. In general, the majority of the active ingredients of traditional chemical pesticides are hydrophobic [32]. Similarly, encapsulating these ingredients of pesticides in nanoparticles is superior to traditional formulation: the dispersion and stability of active ingredients has effectively improved [33, 34]. Nanoparticles could also increase the coverage, adhesion, and penetration of the active ingredients [35].

Lipid, a general term for fats and lipids, belongs to the three categories of nutrients, with the advantages of being renewable and biodegradable, so they are an ideal delivery system for active ingredients [36]. More specifically, the hydroxyl groups in the lipid structure are lipophilic, while the ester chain are hydrophilic; such an amphiphilic structure endows lipids with the potential to be utilized in the delivery system of carrier material [37]. Nanoliposomes, consisting of lipid nanomaterials, are one of the potential options considered in the delivery system of pesticides. Because they have a larger specific surface area and greater solubility and bioavailability than traditional materials, nanoliposomes are regarded as a promising carrier material [38]. With lecithin working as an amphiphilic compound and cholesterol as a shape stabilizer, nanoliposomes have been prepared through ultrasonic homogenization technology by Bang to encapsulate etofenprox [39]. The size of liposome could be reduced to 150 nm by adjusting the proportion of lecithin. To date, however, the use of antiviral agents delivered via nano delivery system to control plant virus diseases has not been reported. This study attempts to develop a nanoliposomes biopesticide delivery system aimed at the control of plant virus diseases in which nanoliposomes are utilized as a carrier to encapsulate quercetin, the environment-friendly antiviral agent.

In this study, quercetin nano-liposome (H-TQ-NPs) was successfully made by thin-film ultrasonic method. This process selected quercetin, lecithin and cholesterol as materials, Tween 80 as the surfactant and hydroxypropyltrimethyl ammonium chloride chitosan (HACC) as the stabilizer. The morphology, particle size, polydispersity index (PDI), zeta potential and encapsulation efficiency of H-TQ-NPs were determined. Then the structure changes of the liposome before and after modification were studied by Fourier transform infrared spectroscopy (FTIR) and transmission electron microscopy (TEM), and the stability of H-TQ-NPs was studied under different conditions. In this study, with Nb-TMV as the research model, the mechanism of quercetin was also explored: the inhibition effect of free quercetin and H-TQ-NPs on TMV were compared at the gene and protein levels. Thus, the antiviral activity of H-TQ-NPs was verified at the individual level. Finally, the efficacy of two formulations of quercetin against TMV was compared in the field.

## Materials and methods

### Materials

Anhydrous quercetin (99%), Dimethyl sulfoxide (DMSO), methanol, trichloromethane and tween-80 were purchased from China National Pharmaceutical Group Co., Ltd. Phosphate buffer (PBS), cholesterol and lecithin were got from Beijing Solarbio Science & Technology Co., Ltd. Chitosan quaternary ammonium salt was purchased from Shanghai yuanye Bio-Technology Co., Ltd. An 8% Ningnanmycin soluble concentrate (8% NL) was purchased from Deqiang Biological Co., Ltd, a 5% Aminooligosaccharin soluble concentrate (5% AL) was purchased from Hainan Zhengye Zhongnong Hi-Tech Co., Ltd, and a 0.5% Lentinan soluble concentrate (0.5% LL) was purchased from Beijing Yanhua Yongle Biological Technology Co., Ltd.

### Preparation and characterization of H-TQ-NPs

**Fig. 1:**
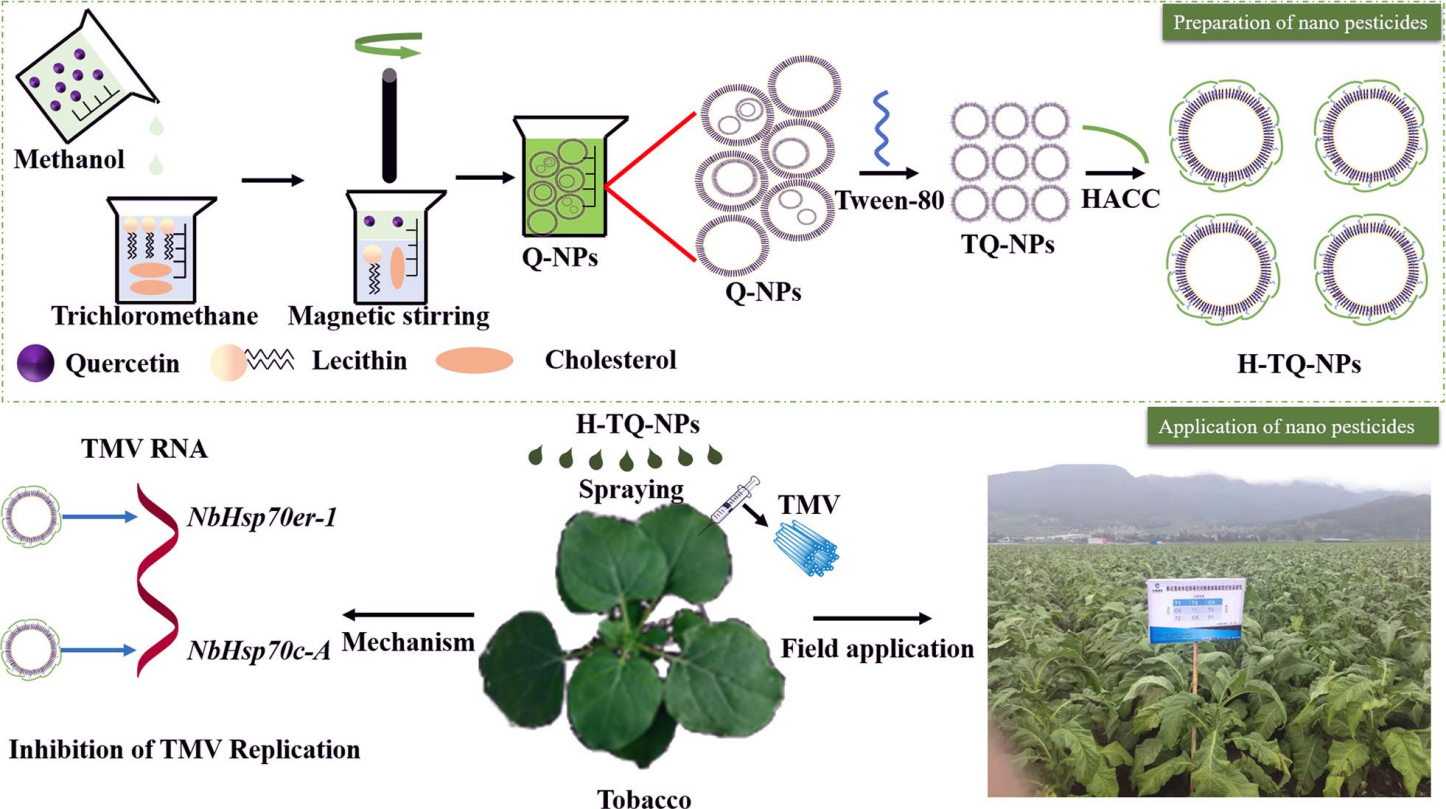
Schematic illustration of the H-TQ-NPs. These can encapsulate the hydrophobic quercetin to control plant virus diseases, the mechanism of this control was investigated, and the control efficiencies of H-TQ-NPs and free quercetin in the field were compared

Anhydrous quercetin (10 mg) was accurately weighed and then dissolved in a 10 mL methanol solution with magnetic stirring for completely dissolution (220 rpm); lecithin (100 mg) and cholesterol (20 mg) were dissolved in 20 ml trichloromethane and magnetic stirring (220 rpm). The methanol solution of quercetin was introduced into the trichloromethane solution, Tween 80 (0.2 mL) was added, and the solute was uniformly dispersed via magnetic stirring. The solution was dried by means of rotation evaporation; the appropriate amount of deionized water was added to dissolve the solute, and a light-yellow film was obtained. After 30 min of setting, an aqueous solution of H-TQ-NPs was obtained through ultrasonic dispersion. HACC solution (0.5 mg/mL) was prepared and diluted, and then the HACC solution was mixed with the H-TQ-NPs solution in a volume ratio of 1:2. The microstructure of H-TQ-NPs was observed by TEM and its particle size, PDI, Zeta potential were determined (Fig. 1).

### Loading efficiency of H-TQ-NPs

The loading efficiency of H-TQ-NPs was determined by indirect method [40]. Firstly, the newly prepared turbid H-TQ-NPs suspension emulsion was centrifuged at high speed. After centrifugation, quercetin was wrapped in nanoliposomes and precipitated at the bottom of the solution, and the free quercetin was dissolved in the supernatant, and 2 mL of the supernatant was extracted with a pipette. The concentration of free quercetin in the supernatant was determined by ultraviolet spectrophotometer at 371 nm. The loading efficiency was calculated according to the following formula (1):

1 $${\text{Loading}}\,{\text{efficiency }}\,{\text{of }}\,{\text{H - TQ - NPs = }}\frac{{{\text{Total }}\,{\text{quercetin - Free }}\,{\text{quercetin}}}}{{{\text{Total }}\,{\text{quercetin}}}}{{ \times 100}}$$

### Stability test

The prepared H-TQ-NP solutions (HACC 0.3 g/L, pH 6.0) were placed separately at different temperatures, and samples were removed regularly to determine the zeta potential, particle size, PDI and quercetin concentration changes at 1 day and 20 days. Then, the pH value of the H-TQ-NPs was adjusted with newly prepared 1 M HCl and 1 M NaOH. The solutions were set for a period, and samples were removed regularly to measure the zeta potential, particle size, PDI and quercetin concentration changes.

### Virus inoculation

The upper new leaves of ordinary tobacco inoculated with TMV were ground with PBS buffer (0.01 M, pH 7.2–7.4) and filtered. The filtrate was centrifuged at 10,000 × *g* for 20 min, and the obtained supernatant was centrifuged at 10,000 × g for 30 min and then resuspended to precipitate with 0.01 mol/L PBS buffer (pH 7.2–7.4) to obtain a TMV inoculation solution [41].

A plasmid containing TMV-GFP was transferred into the agrobacterium GV3101 by the CaCl_2_-mediated freeze–thaw method. Agrobacterium cells were placed in LB solid medium (50 µg/ml kanamycin and 100 µg/ml rifampicin) and cultured at 28 ℃ for 48 h. A single colony of agrobacterium was verified by PCR and then placed in LB liquid medium (50 µg/ml kanamycin and 100 µg/ml rifampicin) at 28 ℃ and 200 rpm until its OD_600_ reached 1.0–2.0. The OD_600_ of the cell solution was adjusted to 0.3–0.5 with an infiltrating solution (10 mM MgCl_2_, 1 mM 2-(*N*-morpholino) and 0.15 mM acetosyringone). Finally, the resuspended agrobacterium infiltrating solution was placed at 28 °C for 3 h to perform the inoculation experiment.

Nb tobacco blades at the 6 to 8 leaf stage were evenly sprinkled with 60-mesh-sieved quartz sand, and the obtained virus inoculation solution was evenly coated on the leaf surface with cotton swabs. Two hours after inoculation, the quartz sand on the surface of the leaves was rinsed with sterile water.

### Abiotic stress treatment

After normal culture at 25 ℃, Nb was cultured in an incubator at 42 ℃ with artificial light for 2 h. The test leaves and normal leaves were stored at − 80 ℃, and the experiments were repeated three times. The relative expression of *Nbhsp70* mRNA and *hsp70* protein was determined.

Before being placed into the incubator at 42 ℃, five groups of Nb were treated as follows: sprayed with water (CK), sprayed with no-load nanoliposomes (K-NPs), sprayed with quercetin solution (QT), soaked in H-TQ-NP solution, and sprayed with H-TQ-NP solution. Three hours after the treatment, these five groups were placed in incubators at 42 ℃ with light for 2 h. The leaves were stored at -80 ℃, and the experiments were repeated three times. The relative expression of *Nbhsp70* mRNA and *hsp70* protein was determined.

### Pot experiment

A certain amount of Nb inoculated with TMV or TMV-GFP was sprayed with H-TQ-NPs, CK, K-NPs, and QT twice at an interval of 12 h, and the experiments were repeated three times. The distribution and quantity of TMV-GFP in Nb was observed regularly. After 60 h of TMV inoculation, the leaves were collected and stored at − 80 ℃, and the relative accumulation of TMV coat protein (TMV-CP) mRNA and the protein expression of TMV-CP were determined.

### Field experiment

Field experiments were carried out on May 21, 2020, in Huiping Green Tobacco Base, Mianning County, Xichang city, Liangshan Prefecture, Sichuan Province. The soil was loam soil with above-average fertility and good drainage and irrigation systems. Three commercially available antiviral agents were selected and used in the control group. A total of five treatment groups with identical concentrations were prepared: H-TQ-NPs, QT, 8% NL, 5% AL and 0.5% LL. The block arrangement was randomized four times. The plant spacing and row spacing were 50 and 110 cm, respectively. During the field experiment, the tobacco was in the root-extended period, which was the initial stage of disease. The incidence rate of plants was 2%, and the highest degree of disease was grade 0. The first pesticide application occurred on June 10, the second application was performed 7 days later, and the third application was performed on July 2 due to the weather.

The application method was spray application with a Xinxiu-16-type electric sprayer, whose working pressure was 3–4 kg/cm^2^ and spray volume was approximately 800 mL/min. The water consumption at three measured times was 225, 300, and 450 L/hm^2^. Five points were randomly sampled in each block, and 6 plants at each point were investigated. The total number of plants and the number of diseased plants at all levels were recorded. Disease progression was investigated before application, and the control efficiency was investigated 7 days after each application. Furthermore, the growth of each treated tobacco was observed to determine whether there was any drug injury.

### Statistical analysis

The least significant difference method (LSD) and Q test method in SPSS software were applied to analyze the significance of the differences in the data from the laboratory experiment. Duncan's new multiple range method (DMRT) was used to analyze the significance of the control effect in the field experiment. All graphical data are reported as the mean ± standard deviation (SD). Significance levels were set at *p < 0.05.

## Results

### Preparation and characterization of H-TQ-NPs

**Fig. 2:**
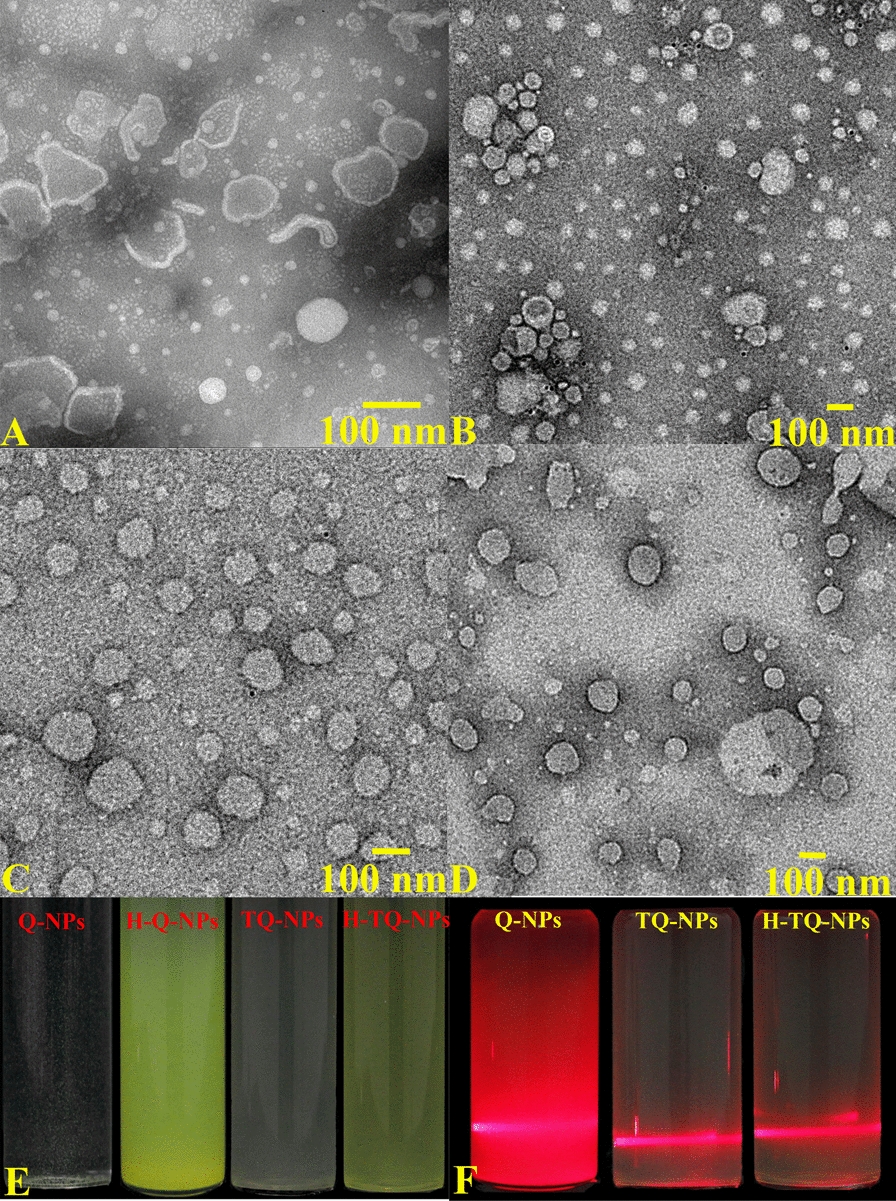
TEM images of Q-NPs (**A**), H-Q-NPs (**B**), TQ-NPs (**C**), and H-TQ-NPs (**D**). From left to right, the appearance of H-TQ-NPs **E** and the Tyndall phenomenon of H-TQ-NPs (**F**)

In this study, quercetin nanoliposomes were prepared by a thin-film ultrasonic method. The microstructure of the H-TQ-NPs was observed by TEM. As shown in Fig. 2A, the quercetin nanoliposomes (Q-NPs), the simplest liposome obtained by rotatory evaporation, were multicompartment liposomes with uneven particles with sizes of 100–350 nm, and their double membrane structure was clearly observed. After adding HACC, a small amount of irregular nanoparticles was formed (Fig. 2B). In the image of the TQ-NPs (Fig. 2), the liposome aggregate disappeared, and the nanoliposomes were distributed in a single chamber, with a stable bilayer structure and a reduced particle size. In the H-TQ-NP sample group (Fig. 2D), the nanoparticles were uniformly dispersed, and the nanoliposome size in the field of vision was mostly approximately 100 nm. It was concluded that the addition of HACC and Tween 80 was of great significance for stabilizing the nanoparticle structures. The Q-NP solution was colorless and turbid. The solution appeared yellow after the addition of HACC, and the transparency of the solution decreased after the addition of Tween 80. The final product (H-TQ-NPs) was a light-yellow turbid solution (Fig. 2 ). Figure 2F shows the Tyndall phenomenon of the Q-NP, TQ-NP and H-TQ-NP aqueous solutions. In the Q-NP solution, which was cloudy, the reflection and refraction of light were strong, and the light beam was not obvious. No light scattering occurred in the TQ-NP and H-TQ-NP solutions, and the light beam was clear.

### Stability test

**Fig. 3:**
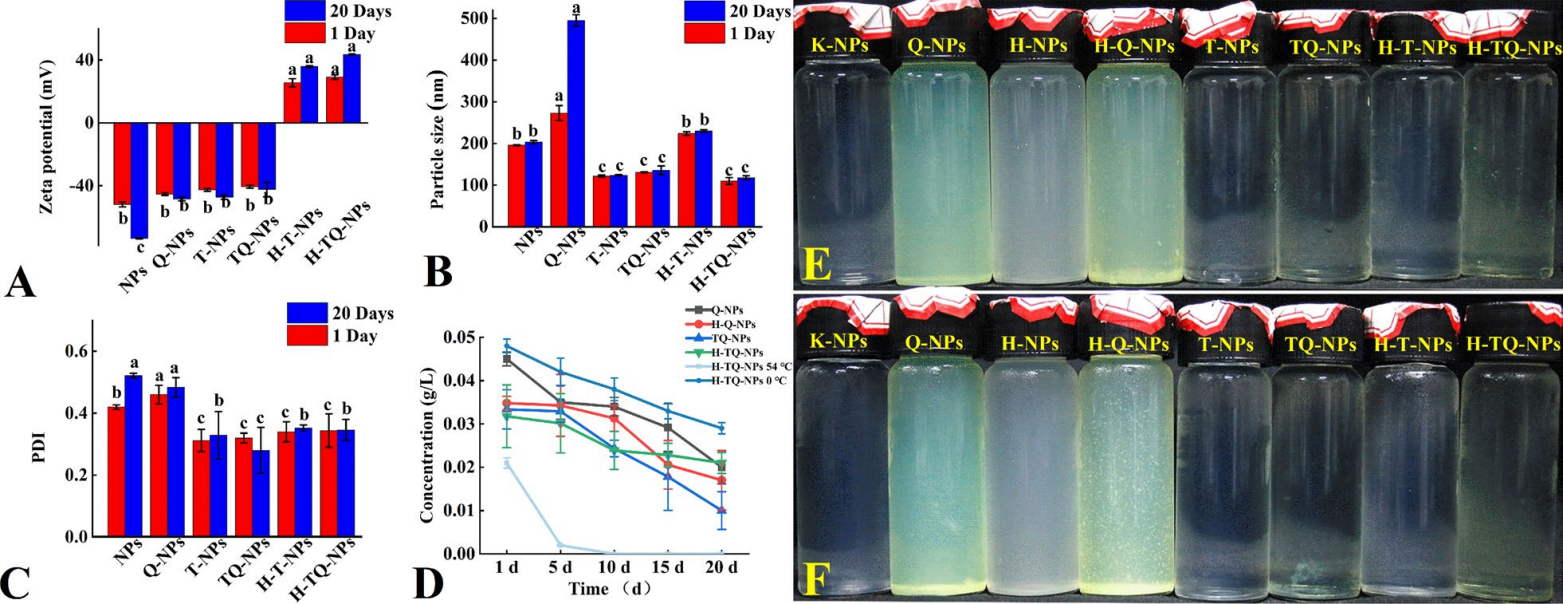
Changes in the zeta potential (**A**), particle size (**B**), PDI (**C**) and quercetin concentration **D** of different treatment groups after 20 days of storage; from left to right, the appearance of H-TQ-NPs **E** after 20 days of storage (**F**); bars with the same letters show no significant differences (LSD test, p < 0.05)

The stability of the H-TQ-NPs was quantitatively characterized by measuring the changes in the zeta potential (Fig. 3A), particle size (Fig. 3B), PDI (Fig. 3 C) and quercetin concentration (Fig. 3D) in each treatment group at 26 ℃ within 20 days. First, the zeta potentials of the newly prepared Q-NPs, TQ-NPs and H-TQ-NPs were − 48.5 mV, − 42.4 mV and + 43.5 mV, respectively, and their absolute values were all greater than 30 mV, suggesting that the particles were stably dispersed in the medium. After 20 days of storage, the absolute value of the zeta potentials of the Q-NPs and TQ-NPs decreased, but that of the H-TQ-NPs supplemented with HACC remained stable, and the absolute value remained greater than 30 mV. Figure 3B shows the particle size changes of each treatment group. The average particle sizes of the newly prepared Q-NPs, TQ-NPs and H-TQ-NPs were 273 ± 10.39 nm, 318.67 ± 12.03 nm, and 117.50 ± 7.11 nm, respectively. After storage for 20 days, the average particle size of the Q-NPs increased to approximately 500 nm, and there was no significant change in the sizes of the TQ-NPs and H-TQ-NPs, which indicated the good stability of the H-TQ-NPs. The changes in PDI of each treatment group are listed in Fig. 3C. The PDI of the newly prepared Q-NPs, TQ-NPs and H-TQ-NPs were 0.48, 0.29 and 0.31, respectively, and changed to 0.55, 0.28 and 0.32, respectively, after 20 days of storage; this lack of a significant change in PDI in each group indicated that the particle distribution of the TQ-NPs and H-TQ-NPs was concentrated and remained stable during long-term storage. Finally, the changes in the concentrations of active ingredients in the system were measured to determine the protective effects of liposomes on quercetin. As shown in Fig. 3D, the quercetin concentrations in the Q-NPs, TQ-NPs and H-TQ-NPs were 43.49 ± 2.85 mg/L, 33.38 ± 1.99 mg/L and 31.76 ± 0.67 mg/L, respectively. After 20 days, the contents of the active ingredients in all systems decreased. The hig hest degree of active ingredient degradation occurred in the TQ-NP treatment group, with an active ingredient content of only 30%; the content in the Q-NP treatment group decreased by 50%; and the H-TQ-NP treatment group had the highest retention of active ingredients, which was more than 70%. At the same time, the stability of H-TQ-NPs at different temperatures (0, 54 ℃) was determined. It could be seen that at 0 ℃, H-TQ-NPs had good stability and the lowest degradation amount of active ingredients, while at 54 ℃, H-TQ-NPs performed poorly. In less than 5 days, the activity ingredients of quercetin were basically completely degraded. This was not only related to quercetin as a biological antiviral agent, we speculate that there was an important reason for the structure of the biomaterial carrier nanoliposome to be destroyed under high temperature conditions. The results showed that the H-TQ-NPs prepared in this study could effectively maintain the structural stability of quercetin and reduce its degradation, which was of great significance for improving the stability of quercetin.

Figures 3E and F show the appearance of each treated aqueous solution on day 1 and day 20, namely, K-NPs, Q-NPs, HACC nanoliposome solution (H-NPs), HACC quercetin nanoliposomes (H-Q-NPs), Tween 80 nanoliposomes (T-NPs), Tween 80 quercetin nanoliposomes (TQ-NPs), HACC Tween 80 solution, and H-TQ-NPs from left to right. The H-TQ-NP solution was clear and transparent with no precipitation, while flocculent precipitates appeared in the H-Q-NP aqueous solution, which was pale yellow. After 20 days of storage, the appearance of the solutions remained basically unchanged: in the H-Q-NP treatment group, the solutes aggregated into large particles; large-particle flocculent precipitates also appeared in the Q-NP and TQ-NP treatment groups; and the H-TQ-NP solution was stable and remained homogeneous and transparent without precipitation

### Loading quercetin

**Fig. 4:**
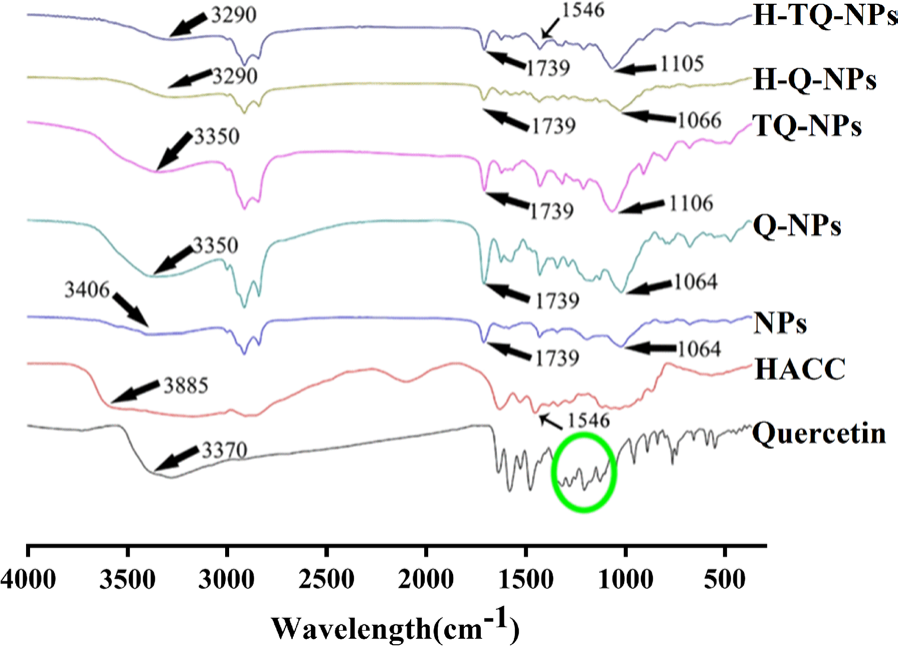
FTIR curves of different quercetin treatment and HACC

The embedding of quercetin was further verified by FTIR analysis of the different treatment groups. In Fig. 4, the characteristic absorption of the sample covered the entire region from 500–4000 cm^−1^. In the range of 500–2000 cm^−1^, the peaks at 1105 cm^−1^ and 1739 cm^−1^ corresponded to the stretching vibration of aromatic hydrocarbons, and the peak at 1546 cm^−1^ corresponded to the stretching vibration of -NH in the amino group. These peaks demonstrated that chitosan had successfully attached to the surface of the liposomes because these groups are characteristic structures of chitosan. The characteristic peak of the stretching vibration of the phenolic hydroxyl group (the characteristic group in the quercetin structure) disappeared in the range of 1200–1400 cm^−1^ compared with that of the original quercetin, which showed that quercetin was successfully encapsulated into the nanoparticles through hydrophobic interactions or hydrogen bonding.

The UV standard curve of quercetin in methanol appeared linear in the range of 0–0.06 mg/mL, and the regression equation was y = 34.429x + 0.074, R^2^ = 0.9989 (Additional file 1: Fig. S1 and Table S1). The concentration of free quercetin in the supernatant was determined to quantify the loading efficiency of the nanoliposomes. The loading efficiency of the TQ-NPs was 82.26% ± 0.30%, and that of the H-TQ-NPs with the addition of quaternary ammonium salt of chitosan was as high as 88.02 ± 0.10%.

### Research on the optimal formula

With the addition of HACC, the stability and loading efficiency of H-TQ-NPs were both improved, and the amount of HACC added was further screened to optimize the formula. Different concentrations of HACC influenced the zeta potential (Additional file 1: Fig. S2A) and PDI (Additional file 1: Fig. S2C): with increasing HACC concentration, the absolute values of the zeta potential and PDI both showed an increasing trend; the addition of HACC enhanced the dispersion of the nanoparticles and expanded their particle size range (Additional file 1: Fig. S2B). The smallest particle size was observed when the HACC concentration was 0.2 g/L, and then it increased as the HACC concentration increased. After 20 days, the quercetin content in the 0.3 g/L treatment group decreased the least (Additional file 1: Fig. S2D). Based on the comprehensive results, the optimal formula contained 0.3 g/L HACC.

### Stability test of H-TQ-NPs

To study the performance of the H-TQ-NPs under different conditions, a series of pH, temperature and light conditions were set. In Additional file 1: Fig. S3, the color of the solution gradually deepened with increasing pH. The solution exhibited slight turbidity when the pH exceeded 6.0, which became more obvious as the pH continued to increase. The zeta potential, particle size, PDI and concentration of active ingredients at different pH values were measured. As shown in Additional file 1: Fig. S4A, with increasing pH value, the zeta potential exhibited a downward trend. Although the zeta potential decreased slightly, its absolute value was over 30 mV, regardless of whether it had been stored for 1 day or 20 days. After the pH increased, the particle size of the H-TQ-NPs gradually increased (Additional file 1: Fig. S4B), especially when the pH was greater than 6.5. Unlike the particle size, the PDI gradually decreased as the pH increased (Additional file 1: Fig. S4C). When the pH exceeded 5.0, the PDI tended to stabilize. After storage for 20 days, the concentration of quercetin in all groups decreased (Additional file 1: Fig. S4D), and the decrease was most obvious at pH 4.0. At pH 7.0, the amount of quercetin degradation was the least. In conclusion, the H-TQ-NPs had good stability in the pH range of 5.0–7.0, and at pH 6.0, the H-TQ-NPs were the most stable.

Next, the influence of different temperatures and light conditions on the H-TQ-NPs was explored (Table 1, Additional file 1: Fig. S5). First, the degradation results showed that light could significantly reduce the content of quercetin, some of which was degraded. Therefore, a low temperature without light was more suitable for the preservation of the H-TQ-NPs (Additional file 1: Fig. S5A). With increasing temperature, the zeta potential, particle size and PDI of the H-TQ-NP solution all decreased, which might be due to the influence of increasing temperature on the loading capacity of HACC. Additionally, the light conditions effectively reduced the particle size of the H-TQ-NPs, while zeta potential and PDI remained in a good state. Temperature and illumination had little influence on the appearance of the H-TQ-NP solution. There was no obvious change in the H-TQ-NPS solutions with different treatments, and the solution remained clear and transparent, without any precipitation (Additional file 1: Fig. S5C).

**Table 1:** Zeta potential, particle size, and PDI of the H-TQ-NPs under different conditions

	Sunlight, 20 ℃	Dark, 4 ℃	Dark, 20 ℃	Dark, 30 ℃	Dark, 40 ℃
Zeta Potential (mV)	34.63 ± 0.81	30.72 ± 0.37	28.64 ± 1.68	29.94 ± 0.28	28.71 ± 1.43
Particle Size (nm)	110.35 ± 2.44	116.85 ± 2.37	124.82 ± 2.71	125.62 ± 1.81	127.19 ± 3.64
PDI	0.45 ± 0.03	0.46 ± 0.04	0.39 ± 0.03	0.41 ± 0.05	0.39 ± 0.02

### Mechanism of control TMV

**Fig. 5:**
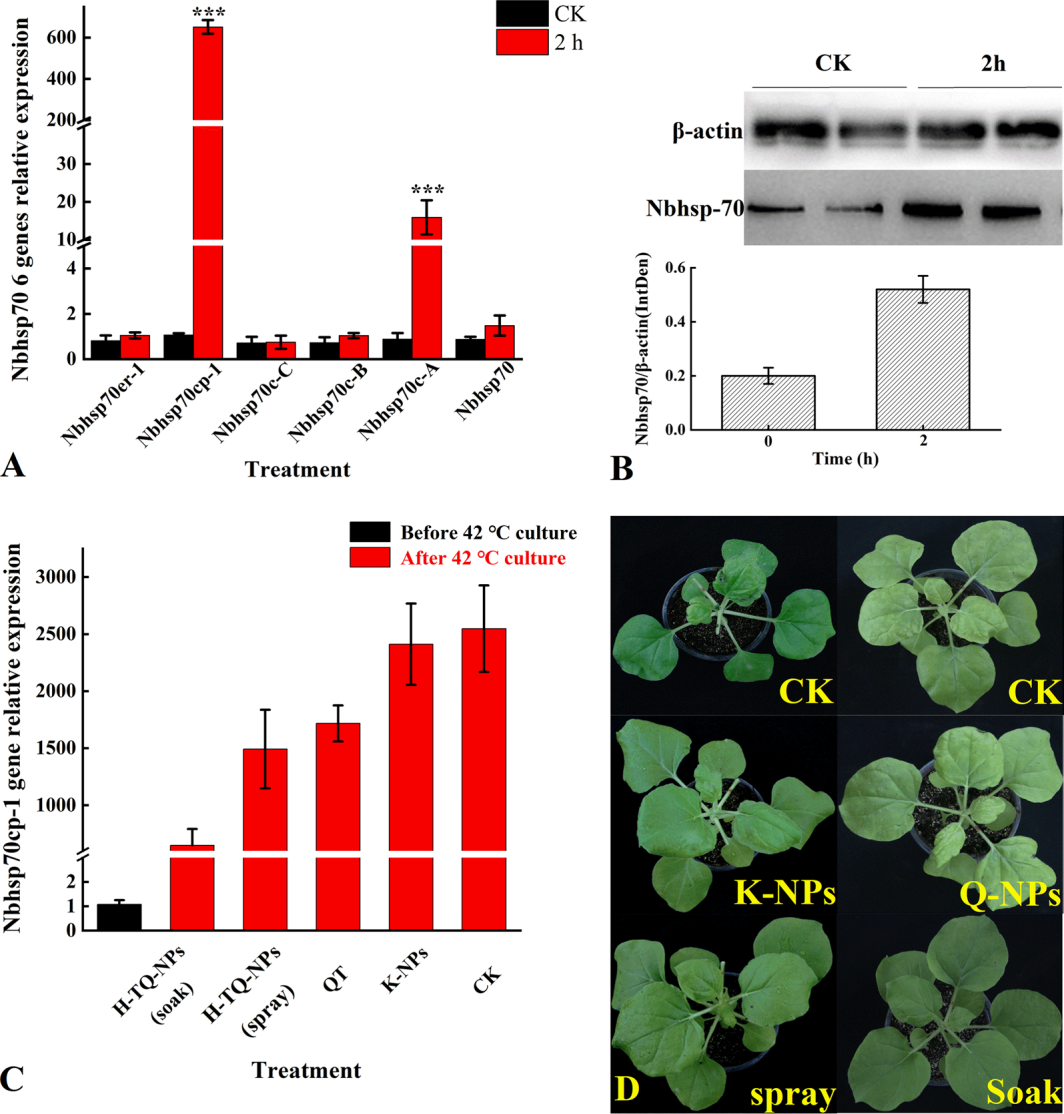
Relative Nbhsp70 gene expression after heat treatment (**A**), western blot of Nbhsp70 protein before and after treatment for 2 h at 42 ℃ on Nb (**B**), relative gene expression of Nbhsp70 treated with different quercetin solutions (**C**), and the appearance of Nb leaves treated with different formulations of quercetin after heat treatment (**D**); bars with the same letters show no significant differences (LSD test, p < 0.05)

The function of the plant hsp70 protein is similar to that of the animal hsp70 protein, which is highly expressed in response to stress. Previous studies have shown that Nbhsp70 plays a role in the process of virus inoculation in plants. Figure 5A shows that after heat treatment, the relative expression levels of *Nbhsp70cp-1* and *Nbhsp70c-A* in the plants increased by 650 times and 16 times compared to CK treatment, respectively. The results of gray analysis of the protein quantification showed that the relative expression levels of the Nbhsp70 protein increased by 2.6 times after heat treatment (Fig. 5B). The relative expression level of *Nbhsp70* in Nb was measured after treatment with different concentrations of quercetin before heat treatment. The results are shown in Fig. 5 C. Compared with that int eh CK group, the relative expression level of *Nbhsp70cp-1* in the H-TQ-NP-soaked group decreased by 74%, and that in the sprayed group decreased by 39%, which indicated that under the two application methods, quercetin could effectively inhibit the expression of *Nbhsp70,* and this effect was more obvious in the soaked group. The individual plant test also showed that the plants were scorched and shrunken after heat treatment, and these symptoms could be significantly reduced when the leaves were soaked and sprayed with the H-TQ-NPs (Fig. 5D). Second, the symptoms of the leaves after QT treatment were also relieved but not as obviously as those of the H-TQ-NP treatment group. The leaves after K-NP treatment still showed symptoms of shrinkage and scorching, and there was no therapeutic effect.

**Fig. 6:**
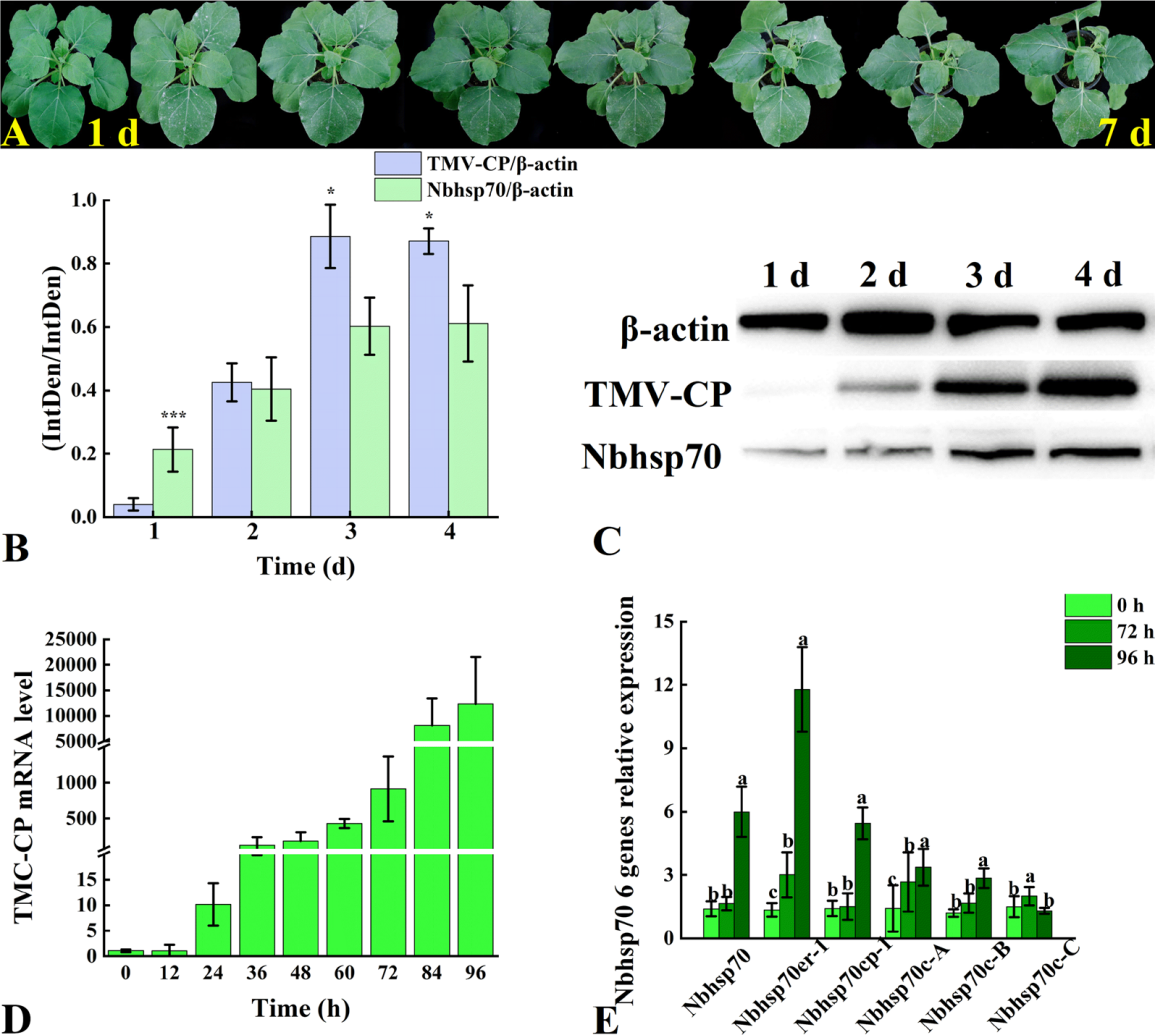
The appearance of Nb inoculation with TMV at 1 day to 7 days (**A**), the relative expression of TMC-CP and Nbhsp70 protein (**B**), western blot (**C**), the relative gene expression of TMV-CP mRNA after inoculation with TMV for 96 h (**D**), and the relative gene expression of Nbhsp70 treated with different quercetin solutions (**E**); bars with the same letters show no significant differences (LSD test, p < 0.05)

After inoculation with TMV, the heart leaves of the Nb underwent obvious shrinkage and curling; the plant grew slowly, and the leaf margin of the whole plant rolled down. Five days later, the tender stem at the growth point of the heart leaves tipped to one side (Fig. 6A). The results of western blot analysis indicated that TMV-CP protein accumulation gradually increased after inoculation, and the protein expression of *Nbhsp70* also increased significantly with time (Fig. 6B, C). In the first 2 days of inoculation, the expression levels of TMV-CP protein and Nbhsp70 protein were basically the same, and after 2 days, the levels increased, with the expression level of TMV-CP protein increasing more significantly. After that, the relative accumulation of TMV-CP in the inoculated leaves was detected by qRT-PCR. At 36 h after inoculation, the relative accumulation of TMV-CP was upregulated more than one 100-fold and increased by more than 15,000 times after 96 h (Fig. 6D). After TMV inoculation for 72 h, the relative expression levels of *Nbhsp70er-1* and *Nbhsp70c-A* were both increased by approximately twofold (Fig. 6E). After 96 h, the expression of *Nbhsp70er-1* still showed the most significant change, with an increase of nearly 20 times. In addition, the relative gene expression levels of *Nbhsp70* and *Nbhsp70cp-1* increased by approximately fourfold.

**Fig. 7:**
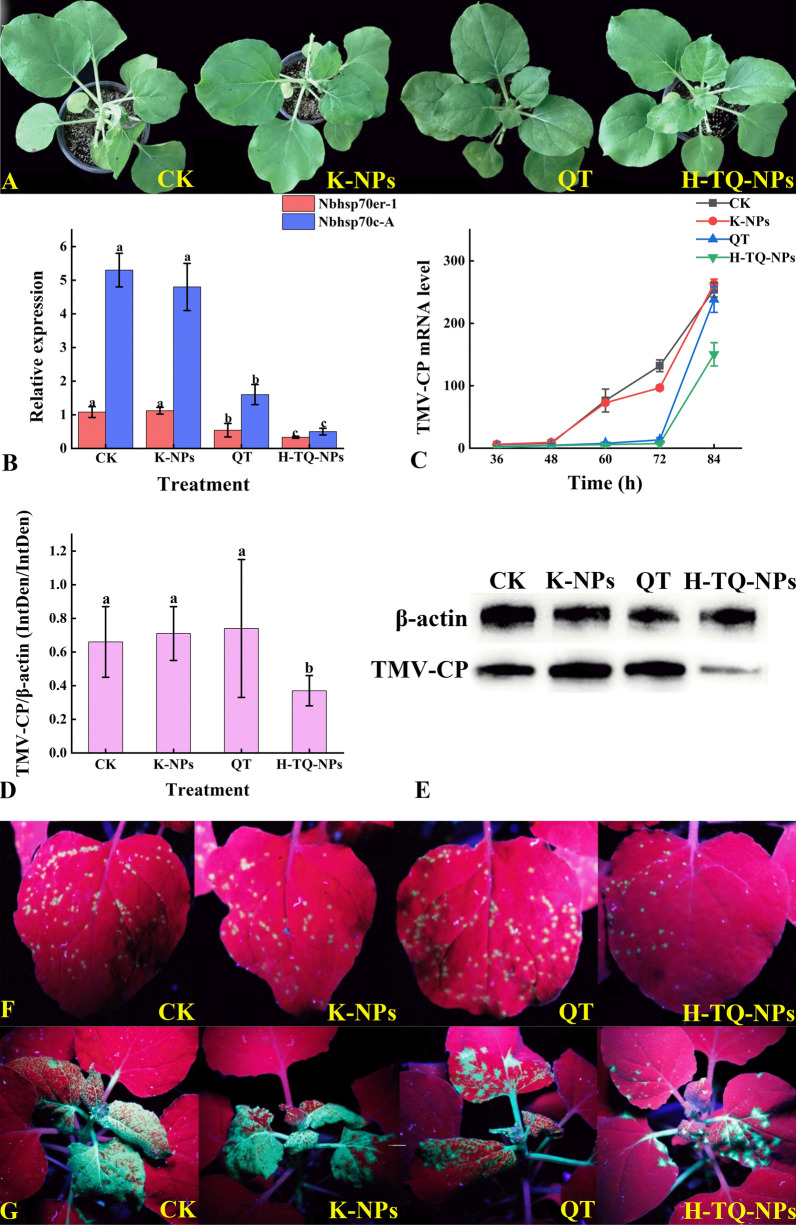
The appearance of inoculated Nb leaves (**A**), the relative gene expression of Nbhsp70er-1 and Nbhsp70c-A (**B**), the change in TMV-CP mRNA relative gene expression (**C**), the relative expression of TMV-CP treated with different quercetin solutions (**D**), western blot (**E**), and the appearance of Nb leaf inoculation with TMV-GFP on 5 days (**F**) and 8 days (**G**) treated with different quercetin solutions; bars with the same letters show no significant differences (LSD test, p < 0.05)

Consistent with abiotic stress treatment, TMV inoculation also resulted in upregulated expression of *Nbhsp70cp-1* and *Nbhsp70c-A*, which means that *Nbhsp70cp-1* and *Nbhsp70c-A* were the response factors of the host plants during TMV inoculation. Then, the plants inoculated with TMV were treated with quercetin, and the changes in the relative expression levels of *Nbhsp70er-1* and *Nbhsp70c-A* were determined by qRT-PCR. The relative expression levels of *Nbhsp70er-1* and *Nbhsp70c-A* after quercetin treatment were reduced by 49% and 72%, respectively, compared with those in the CK group (Fig. 7B). These results demonstrated that the upregulation of *Nbhsp70er-1* and *Nbhsp70c-A* was inhibited by quercetin treatment. The proliferation of TMV was inhibited by downregulating the expression of the two genes, thus achieving control of TMV. Therefore, increasing the transport and penetration efficiency of quercetin in plants is of great significance for improving the control efficiency (Table 2).

**Table 2:** The control efficiency of the H-TQ-NPs and other antiviral pesticides in pot experiment; bars with the same letters show no significant differences (LSD test, p < 0.05)

	Initial disease index	Disease index	Control efficiency (%)
QT	6.23	40.32	52.89c
8% NL	5.86	28.62	64.45b
5% AL	6.15	26.28	68.89b
0.5% LL	5.64	25.27	67.38b
H-TQ-NPs	6.15	22.51	73.35a
CK	6.38	87.64	–

### Pot experiment

Nanotechnology was applied to encapsulate the active ingredients in nano delivery systems. The nanometer size not only improved the solubility of the active ingredients but also enhanced the transport and penetration efficiency. To determine whether the H-TQ-NPs prepared in this study could improve the efficiency of quercetin in controlling plant viral diseases, gene, protein, and individual plant tests were carried out. First, the expression levels of *Nbhsp70er-1* and *Nbhsp70c-A* in the inoculated tobacco plant treated with different formulations of quercetin were decreased, while the relative expression levels of the two genes in the unloaded quercetin liposome group were consistent with those of the control group, which did not show an inhibitory effect. Compared with that of the free quercetin treatment, the inhibition effect of the H-TQ-NPs on *Nbhsp70er-1* was 40.8% higher, and the inhibition effect on *Nbhsp70c-A* was increased by 26.4% (Fig. 7B). The H-TQ-NPs could effectively improve the inhibitory effect of the active ingredients on the two target genes to inhibit TMV reproduction. Because the relative expression of *Nbhsp70er-1* and *Nbhsp70c-A* was inhibited, the replication process of TMV in plants was also inhibited. The inhibitory effect of the preparations on TMV was further compared by measuring the accumulation of TMV-CP in inoculated plants. After treatment for 72 h, the relative expression of TMV-CP mRNA in the free quercetin and H-TQ-NPS groups was reduced by 83% and 93%, respectively (Fig. 7C). Compared with that in the control group, the accumulation of TMV-CP was better inhibited after treatment with the H-TQ-NPs. Then, the protein accumulation of TMV-CP was analyzed. Although free quercetin treatment also downregulated the relative expression of *Nbhsp70er-1* and *Nbhsp70c-A* and reduced the accumulation of TMV-CP, the relative accumulation of TMV-CP protein in the free quercetin treatment group was not significantly different from that in the control group. However, the relative accumulation of TMV-CP protein in the H-TQ-NP treatment group was 42% less than that in the control group, which indicated the inhibitory role of H-TQ-NPs on TMV at the protein level (Fig. 7D, E). The experimental results of pot experiment show that several antiviral agents had a certain control effect on TMV in an indoor environment. It could be seen that the control effect of the unprocessed QT solution on TMV was the worst, with a control effect of 52.89%, which may be caused by poor water solubility. Secondly, the effect of several commercial antiviral agents was basically close. And the best control effect was the H-TQ-NPs prepared in this study, the control effect reached 73.35%, which indicated that after preparing nanoliposomes to improve the water solubility, the improvement of the control effect was also more obvious.

To study the effects of quercetin on the proliferation of TMV more intuitively, Nb was inoculated with TMV-GFP, which exhibited green fluorescence, and then water, K-NPs, free quercetin and H-TQ-NPs were sprayed on the leaves. The quantity and distribution of TMV-GFP were observed under ultraviolet light. After TMV-GFP inoculation for 5 days (Fig. 7F), the distribution of TMV-GFP green fluorescence in the inoculated leaves under UV light was poor after H-TQ-NP treatment, followed by free quercetin treatment, which indicated that quercetin could inhibit the proliferation of TMV-GFP at the initial site of virus inoculation, and the inhibitory effect of quercetin nanoliposomes was higher than that of free quercetin. Furthermore, the fluorescence accumulation of TMV-GFP in the K-NP group without quercetin was similar to that in the control group, showing no inhibitory effect on the proliferation of TMV-GFP. After 8 days (Fig. 7G), the TMV-GFP distribution was dense in the control group, with high green fluorescence intensity and severe leaf deformity in comparison with those in the quercetin-treated group, where the TMV-GFP distribution was relatively low, the green fluorescence was sparse, and the leaf deformity was mild. The TMV-GFP distribution was the lowest in the H-TQ-NP-treated tobacco leaves. These results intuitively indicated that the H-TQ-NPs effectively reduced the initial proliferation of TMV-GFP in inoculated leaves, thereby reducing the amount of TMV-GFP and delaying the onset.

### Field experiment

**Fig. 8:**
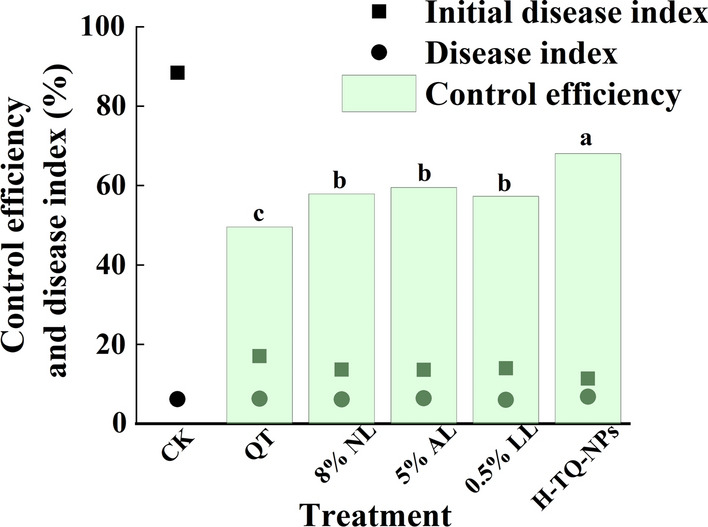
The control efficiency of the H-TQ-NPs and other antiviral pesticides in the field; bars with the same letters show no significant differences (LSD test, p < 0.05)

Finally, the control efficiency of each formulation of quercetin against TMV was researched in the field (Fig. 8); its safety on tobacco plants compared with that of common antiviral agents was verified: from the beginning of the experiment to the tobacco harvest, no phytotoxicity phenomena were observed; the growth of each plant was normal, and the treatment was safe for the growth of tobacco within the range of the test dose. At the same time, the field experiment results showed that all five formulations could play a certain role in controlling TMV. Among them, H-TQ-NPs showed the best control efficiency, with an average rate of 68.08%, which was significantly superior to those of the other three control formulations, namely, 5% for AL, 8% for NL and 0.5% for LL, with average control efficiencies of 59.52, 57.91 and 57.31%, respectively. The lowest control rate was observed for the quercetin conventional formulation, with a value of 49.55%.

## Discussion

Recently, nanotechnology has become increasingly widely used in agriculture, especially in the production of crops; nanocarriers can be used to overcome environmental and biological obstacles to agricultural production and efficiently deliver active ingredients to destinations to improve the control efficiency on target organisms [42]. At present, what is used for delivery is not limited to traditional pesticides but also includes double-stranded RNA, viral DNA, Bt toxin, and so on to protect crop production [43–45]. Previous studies have shown that the application of quercetin for plant protection is associated with favorable antibacterial and antiviral activities. Therefore, nanoliposomes were used as carriers to deliver quercetin. There have been many studies using nanoliposomes as carriers for the delivery of herbicides, insecticides and other traditional pesticides [46]. In this study, the biomaterials lecithin and cholesterol with low cost and high compatibility were used as carriers; the production cost of the carrier was only US$ 0.5/g. The nanoliposomes were prepared by the thin-film ultrasonic method, and in the final stage of preparation, the organic solvent was removed by rotary evaporation to obtain the product, which not only further reduced the production costs but also eliminated the adverse effects of organic solvents on the environment during applications. Therefore, cost and environmental factors, the nanoliposomes prepared in this study were suitable for the delivery of active ingredients in the field considering. There have been no reports on the use of nanocarriers to deliver plant antiviral agents. The quercetin nanoliposomes prepared in this study for the control of plant virus diseases were investigated for the first time, and their optimal preparation conditions and storage environment were evaluated.

Lecithin and cholesterol were chosen to reduce the allergic reaction of plants to foreign and nonplant substances [47]. The initial embedding of quercetin was achieved as a result of the amphiphilicity of the phospholipid group, and the addition of Tween 80 helped to improve the interface stability of the liposomes and increase the amount of nanoliposomes attached to the surface of plant leaves. The permanent positive charge of the quaternary ammonium group in the HACC could increase the electrostatic interaction in the lecithin emulsions and the electrostatic repulsion in the droplets, thereby avoiding the aggregation of liposomes [48]. The H-TQ-NP solution was transparent without precipitation after the addition of HACC and Tween 80, the nanoparticles in the TEM image were uniformly dispersed, and the Tyndall effect was the most obvious for the H-TQ-NPs. A radius of dispersed particles less than 100 nm was less than the wavelength of the incident light, resulting in scattering and allowing the light column to be clearly seen. The particle size, zeta potential and PDI also confirmed this phenomenon, and the particle size was significantly reduced after adding HACC and Tween 80. Many properties, including water solubility, permeability, adhesion and so on, could be improved by preparing traditional chemical pesticides as nanoscale particles [49]. At the same time, the zeta potential of the nanoliposomes exceeded 50 mV, which meant that the particles had good dispersibility. PDI is an indicator for evaluating the uniformity of the particle size distribution, and the smaller the value is, the higher the concentration of the particle size distribution. The PDI was reduced by 35.4% compared to that of the Q-NPs; the particle size distribution was more concentrated, and there were basically no particles of other sizes generated. A more stable structure was formed to wrap quercetin, consistent with the TEM images, to effectively protect quercetin.

The best preparation conditions for the H-TQ-NPs at different concentrations of HACC and different pH values were explored, and the H-TQ-NPs prepared under the conditions of 0.3 g/L HACC and pH 6.0 had the best performance. The FTIR results showed that HACC was successfully attached to the surface of the H-TQ-NPs, and the positive charge carried by HACC increased the electrostatic repulsion between the nanoparticles. However, the addition of excessive HACC increased the number of groups attached to the surface and the particle size of the nanoliposomes. The uneven increase in particle size decreased the concentration of the particle size distribution, and the PDI value fluctuated; therefore, adding 0.3 mg/mL HACC led to the most stable performance. Since the surface of the H-TQ-NPs had several positive charges, the H-TQ-NPs were appropriately stabilized under weak acid conditions. Under alkaline conditions, the increase in anions reduced the positive repulsion on the surface and further reduced the stability and dispersibility of the nanoliposomes. Therefore, the solution could be adjusted to be weakly acidic during the preparation and storage of the H-TQ-NPs. Finally, to clarify the storage conditions of the H-TQ-NPs, the influence of different temperature and light conditions on the H-TQ-NPs was researched. The results of the stability test showed that H-TQ-NPs had excellent stability, and there was no significant change in the zeta potential, particle size, or PDI after 20 days of storage. The H-TQ-NPs had excellent stability under low temperature and dark conditions. The release rate was decreased because the activity of the nanoparticles was reduced at low temperature in the dark. At the same time, the quercetin released at low temperature in the dark degraded slowly, so the stability was better.

Quercetin could effectively reduce the number of viruses in the host plant, but studies have also shown that quercetin did not directly affect virus replication and did not induce host resistance [50, 51]. As a heat shock protein, the hsp70 protein is expressed in large quantities during biotic and abiotic stress and participates in protein assembly, transportation and degradation [52, 53]. The distribution of the *hsp70* gene in the nucleus, cytoplasm, mitochondria, chloroplasts and endoplasmic reticulum has long been reported [54]. The viral replication complex is assembled from viral proteins and viral genomic RNA and necessary host factors [55], and it preferentially accumulates in the membrane structure of organelles such as the endoplasmic reticulum and chloroplasts. TMV forms a viral replication complex on the endoplasmic reticulum and may recruit *Nbhsp70er-1* to participate in the folding of viral proteins. Under heat stress, the gene and protein expression of *Nbhsp70cp-1* and *Nbhsp70c-A* in Nb was upregulated, and quercetin treatment inhibited *Nbhsp70cp-1* and *Nbhsp70c-A* expression. Therefore, we speculated that the mechanism of quercetin against TMV also involved inhibiting the expression of the *hsp70* gene. After Nb inoculation with TMV, the plants grew slowly, the leaves shrank and curled, the leaf edge curled down, the accumulation of TMV-CP and TMV-CP increased, and the relative expression of *Nbhsp70er-1* and *Nbhsp70c-A* was upregulated. The treatment of inoculated plants with quercetin validated our conjecture that quercetin inhibited viral activity by downregulating the relative expression of *Nbhsp70er-1* and *Nbhsp70c-A*.

The inhibition mechanism of quercetin was clarified, and it was considered that the low water solubility of quercetin was an important factor limiting the control effect. Based on the nanoscale particle size and amphiphilic nature of liposomes, the carrier could efficiently deliver quercetin to the inside of cells through osmosis or endocytosis. Research has shown that chitosan-coated liposomes were Higuchi models [56], indicating that the H-TQ-NPs prepared in this study permeated into the cell through penetration. The nanoparticles were destroyed by lipase in the cell, and quercetin could be released to reach the organelles. As expected, compared with free quercetin, the H-TQ-NPs increased the inhibitory effect of quercetin on *Nbhsp70er-1* and *Nbhsp70c-A*, thereby inhibiting the replication of TMV and reducing the accumulation of TMV-CP mRNA and TMV-CP. Finally, the incidence of intact plants inoculated with TMV under laboratory conditions was significantly reduced, and the expression and distribution of fluorescent TMV-GFP was reduced. The field experiment also proved that the development of quercetin into nanoparticles had a better control effect than free quercetin. The nanoliposomes significantly improved the solubility of quercetin, which enabled efficient transport into plants and entrance into cells through penetration, thus inhibiting the expression of *hsp70* protein and ultimately improving the prevention of TMV.

The preparation of high-efficiency and environmentally friendly antiviral agents to control plant virus diseases is of great significance to agricultural production and development. As a plant-derived agricultural antiviral agent, quercetin is widely used for plant protection. In previous studies, we verified that quercetin could be used for the control of TMV, but it had a short duration and was easy to degrade; the inhibitory effect on TMV was less than 7 days, so quercetin could be delivered into cells to exert its effect in a short time. However, the H-TQ-NPs prepared in this study significantly improved the transportation and delivery efficiency of quercetin, making the control efficiency on intact plants in the laboratory or in the field environment higher than that of free quercetin. In the future, H-TQ-NPs will continue to be used on a large scale in the field, and the safety of nanoliposomes should also be considered. The toxicity of lecithin and cholesterol in the plant could be ignored. This was also confirmed by the fact that there was no phytotoxicity in the field environment. Therefore, quercetin nanoliposomes could be considered a potential nano biopesticide to improve the control efficiency of antiviral agents.

## Conclusion

In summary, a nanoliposome carrier was constructed using lecithin and cholesterol as materials and encapsulated the hydrophobic antiviral agent quercetin based on the amphiphilicity of the phospholipid group. The particle size of the quercetin nanoliposomes was controlled at approximately 110 nm, the absolute value of the zeta potential exceeded 30 mV, the PDI was less than 0.3, and the nanoliposomes showed good stability even after 20 days of storage. Quercetin downregulated the expression of hsp70 protein by inhibiting *Nbhsp70er-1* and *Nbhsp70c-A*. hsp70 is a key protein involved in virus replication, and the replication of TMV was influenced, thereby achieving control of TMV. Because of their nanoscale particle size and hydrophilicity, nanoliposomes can efficiently transport quercetin in plants, penetrate the cell membrane through osmosis, and reach the vicinity of the organelle where the target gene exists. Therefore, quercetin nanoliposomes could effectively improve the inhibitory activity of quercetin on TMV. Under indoor conditions, the inhibition rates of quercetin nanoliposomes on the two main target genes increased by 40.8 and 26.4%, and the accumulation of TMV-CP decreased by 42.1%. Compared with that of free quercetin, the control effect was increased by 37.4% in the field environment. This was the first time that a nanodelivery system has been used for the control of plant virus diseases. The application of quercetin nanoliposomes was beneficial to reduce the use of conventional pesticides, and it has broad potential in the control of plant virus diseases.

## Supplementary Information

**Additional file 1.** Material in qRT-PCR, western and field application. Quantitative detection of NbHsp70 gene and TMV-CP gene. **Table S1.** Primer pairs used to detect gene RNA accumulation. Quantitative detection of Hsp70 protein and TMV-CP protein. Methods of disease grading. **Figure S1. **The standard curve of quercetin. **Figure S2.** The zeta potential (**A**), particle size (**B**), PDI **C** and quercetin concentration **D** of H-TQ-NP solutions with different HACC concentrations; bars with the same letters show no significant differences (LSD test, p < 0.05). **Figure S3.** From left to right, the appearance of H-TQ-NP solutions at pH 4.5, 5.0, 5.5, 6.0, 6.5, 7.0, and 7.5 on days 1 **A** and 20 (**B**). **Figure S4.** The zeta potential **A**, particle size **B**, PDI **C** and quercetin concentration **D** of H-TQ-NP solutions at different pH values; bars with the same letters show no significant differences (LSD test, p < 0.05). **Figure S5.** The change in the quercetin concentration **A** under different conditions; from left to right, the appearance of the H-TQ-NP solutions under sunlight at 20 ℃ and in the dark at 4 ℃, 20 ℃, 30 ℃, and 40 ℃ on days 1 **B** and 20; bars with the same letters show no significant differences (LSD test, p < 0.05).

## Data Availability

All data generated or analyzed during this are included in this published article.
