# Supplementary material for: Field application of nanoliposomes delivered quercetin by inhibiting specific hsp70 gene expression against plant virus disease
Source: J Nanobiotechnology. 2022 Jan 4;20:16. doi: 10.1186/s12951-021-01223-6 (PMC8725512; doi:10.1186/s12951-021-01223-6)
Supplement: Supplementary file 1 — Additional file 1. Material in qRT-PCR, western and field application. Quantitative detection of NbHsp70 gene and TMV-CP gene. Table S1. Primer pairs used to detect gene RNA accumulation. Quantitative detection of Hsp70 protein and TMV-CP protein. Methods of disease grading. Figure S1. The standard curve of quercetin. Figure S2. The zeta potential (A), particle size (B), PDI C and quercetin concentration D of H-TQ-NP solutions with different HACC concentrations; bars with the same letters show no significant differences (LSD test, p < 0.05). Figure S3. From left to right, the appearance of H-TQ-NP solutions at pH 4.5, 5.0, 5.5, 6.0, 6.5, 7.0, and 7.5 on days 1 A and 20 (B). Figure S4. The zeta potential A, particle size B, PDI C and quercetin concentration D of H-TQ-NP solutions at different pH values; bars with the same letters show no significant differences (LSD test, p < 0.05). Figure S5. The change in the quercetin concentration A under different conditions; from left to right, the appearance of the H-TQ-NP solutions under sunlight at 20 ℃ and in the dark at 4 ℃, 20 ℃, 30 ℃, and 40 ℃ on days 1 B and 20; bars with the same letters show no significant differences (LSD test, p < 0.05). [file 12951_2021_1223_MOESM1_ESM.docx]

Material in qRT-PCR, Western and Field Expeirment

Glycine (62011519), SurePAGE precast gel (12%, M00656), methanol (1001411801), sodium chloride (10019308) and hydrochloric acid (FA46650001) were purchased from China National Pharmaceutical Group Co., Ltd. Tris-Tricine-SDS-PAGE Loading buffer (P1325) and Tris (Hydroxymethyl) Aminomethane (T8060), PVDF Transfer Membrane (ISEQ00011) were gotten from Beijing Solarbio Science & Technology Co., Ltd. Tween-20 (P1379) was gotten from Beijing Biotopped Science Co., Ltd. Skim Milk Powder was obtained from Yafho Bio-Technology Co., Ltd. Blue Plus Ⅱ Protein Marker (DM111) was obtained from TransGen Biotech Co., Ltd. Eecl Western Blot Kit (CW0049M), Anti β-Actin Mouse Monoclonal Antibody (CW0264M), Plant Protein Extraction Kit (CW0885M) and Plant Protein Extraction Kit (CW0885M) were bought from CWBIO Co., Ltd. The primary antibody of TMV-CP (SRA57400/10) was purchased from American Agdia Corporation. Primary antibody of Heat shock protein 70 (AS09 592) was purchased from Swedish Agrisera Corporation. Goat Anti-Mouse IgG H&L (HRP, ab97023) and Goat Anti-Rabbit IgG H&L (HRP, ab97048) were bought from Abcam (Shanghai) Trading Co., Ltd. Total RNA Isolation Kit (RC101-01), HiScript® III RT SuperMix for qPCR (R323-01) and ChamQ Universal SYBR qPCR Master Mix (Q711-03) were purchased from Vazyme Biotech Co., Ltd.

Nb cultivation

The moss soil was watered and mixed, and was put into a nutrient bowl (25 cm in diameter). Nb seeds were evenly spread on the surface of the soil layer according to twice the required number of plants. After sealing with plastic wrap, it was placed in a 25 ℃ light incubator (light: dark, 16:8). When the seedlings grow out of two cotyledons, the plastic wrap was removed and continue to cultivate for 2-3 days before transplanting. The moss soil and ordinary substrate were mixed in a ratio of 1:1 and watered, the seedlings were dug deeply and moved into a new pot (diameter 12 cm, height 15 cm) together with the substrate near the roots of seedlings. During the transplanting process, pay attention that the roots of the seedlings should not be damaged to increase the survival rate of transplanting, and continue to cultivate under the same conditions, the trace element fertilizer was diluted at a ratio of 1:1000 to fertilize the plants.

Quantitative Detection of Gene *NbHsp70* and TMV-CP

Plant total RNA was extracted via Total RNA Isolation Kit (Vazyme). According to the product specifications (HiScript®III RT Supermix for qPCR, Vazyme), 1 µg of plant total RNA was taken for cDNA synthesis, PCR reaction (Chamq Universal SYBR qPCR Master Mix, Vazyme) was performed with 2 µl cDNA as template. RT‐qPCRs reaction was performed using a real-time qPCR apparatus (Applied Biosystems, 7500). Primer pairs used to detect gene RNA accumulation were detailed in the table below (Table S1).

Table S1 Primer pairs

| Gene | Accession Number | Forward primer（5’-3’） | Reverse Primer（5’-3’） |
| --- | --- | --- | --- |
| *NbHsp70* | KX912913.1 | TACTGCTGGAGACACTCACCTTGG | TGGTGGTCTGAGCGGTGGATG |
| *NbHsp70er-1* | KX912913.1 | AGCCCTAGTAATCCAGAGGAAAGCAG | TCCTGGCCCAGCAGTCTTCTCG |
| *NbHsp70cp-1* | AB181295.1 | ATTCCTCCAGCTCCTCGTGG | TTTCGGCAAAGTACTAGCACCGG |
| *NbHsp70c-A* | AB112814.1 | AGCTTAGCGCGGATGACAAGAAG | CACCAGCATCACCACCAGCAC |
| *NbHsp70c-B* | AB112815.1 | TGCCATTGAGTCAGCCATACAGTG | CCATGTCACCACCTGCACCTTG |
| *NbHsp70c-C* | AB112816.1 | AGGCAATCTCATGGCTTGACAGC | TTCACCACCAGCGCTTTGGTAC |
| *β-Actin* | NM_007393.5 | CAAGGAAATCACCGCTTTGG | AAGGGATGCGAGGATGGA |
| *TMV-CP* | HE818417.1 | GAGTAGACGACGCAACGG | CCAGAGGTCCAAACCAAAC |

Quantitative Detection of *Hsp70* Protein and TMV-CP Protein

Soluble protein samples were extracted from leaf tissues inoculated with virus according to Plant Protein Extraction Kit (CWBIO, CW0885M). Total proteins were isolated with 12% SDS-PAGE gel and then transferred to the PVDF membrane, the anti-TMV-CP antibody (1:2000) and anti-hsp70 antibody (1:10,000) were used as primary antibodies to detect the expression levels of TMV-CP and hsp70 in plants respectively. Goat anti-rabbit immunoglobulin, together with alkaline phosphatase were used as the secondary antibody (1:5000) to detect chemiluminescence. The gray-scale of western blot bands was scanned by QuantityOne software to judge the differences in protein expression.

Methods of Disease Grading in Field Experiment.

Grade 0: no disease in the whole plant;

Grade 1: interior leaves with clear veins or slight mosaic, no obvious dwarfing of the infected plant;

Grade 3: mosaic is found in 1/3 of the leaves, but the leaves are not deformed, or the infected plant dwarfs to more than 3/4 of the normal plant height;

Grade 5: mosaic is found in 1/3-1/2 leaves, or a few leaves are deformed, or the main veins blacken, or the infected plant dwarfs to 2/3-3/4 of the normal plant height;

Grade 7: mosaic is found in 1/2-2/3 leaves, or leaves become deformed, or the main side vein becomes necrotic, or the infected plant dwarfs to 1/2-2/3 of normal plant height;

Grade 9: mosaic is found in the whole plant, leaves are seriously deformed or necrotic, or the infected plant dwarfs to more than 1/2 of the normal plant height.

The disease index should be investigated before application, and after 3 times of application, 7 days later, the index is investigated, then the control efficiency is calculated according to the following formula:

$$\text{Disease Index=}\frac{\text{Σ(Number of Diseased }\text{Leaves×The}\text{ Disease Grade)}}{\text{(The Total Number of }\text{Leaf×The}\text{ Highest Disease Grade)}}\text{×100}$$

$$\text{Correction Control Efficiency}\text{（}\text{\%}\text{）}\text{=}\text{（}\text{1-}\frac{\text{CK0×PT1}}{\text{CK1×PT0}}\text{）}\text{×100}$$

CK0: disease index of CK before application;

CK1: disease index of CK after application;

PT0: disease index of treatment after application;

PT1: disease index of treatment after application.





Figure S1. The standard curve of quercetin.





Figure S2. The zeta potential (A), particle size (B), PDI (C) and quercetin concentration (D) of H-TQ-NP solutions with different HACC concentrations; bars with the same letters show no significant differences (LSD test, p < 0.05).


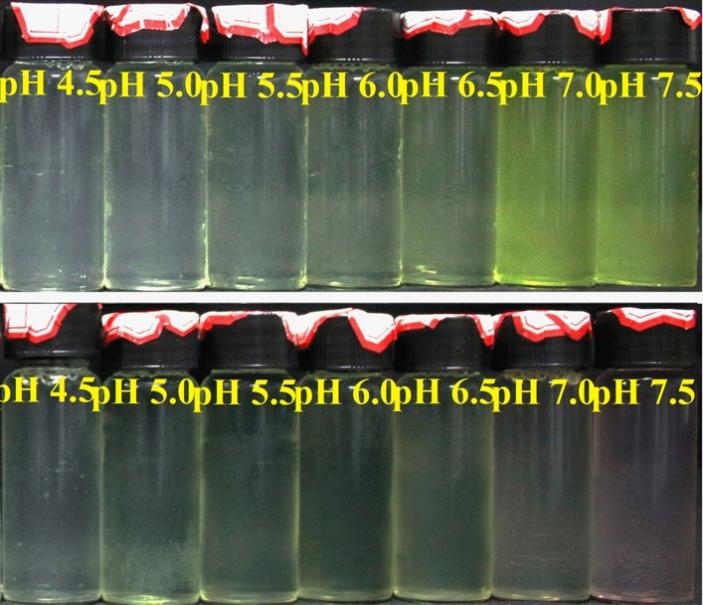


Figure S3. From left to right, the appearance of H-TQ-NP solutions at pH 4.5, 5.0, 5.5, 6.0, 6.5, 7.0, and 7.5 on days 1 (A) and 20 (B).





Figure S4. The zeta potential (A), particle size (B), PDI (C) and quercetin concentration (D) of H-TQ-NP solutions at different pH values; bars with the same letters show no significant differences (LSD test, p < 0.05).





Figure S5. The change in the quercetin concentration (A) under different conditions; from left to right, the appearance of the H-TQ-NP solutions under sunlight at 20 ℃ and in the dark at 4 ℃, 20 ℃, 30 ℃, and 40 ℃ on days 1 (B) and 20; bars with the same letters show no significant differences (LSD test, p < 0.05).
